# Supplementary material for: A high-resolution mRNA expression time course of embryonic development in zebrafish
Source: eLife. 2017 Nov 16;6:e30860. doi: 10.7554/eLife.30860 (PMC5690287; doi:10.7554/eLife.30860)
Supplement: Supplementary file 6. [file elife-30860-supp6.zip › biolayout-clusters-files/Cluster127-genes.html]

Cluster127


# Cluster127: Genes

| Ensembl ID | Gene Name | Chr | Start | End | Biotype |
| --- | --- | --- | --- | --- | --- |
| ENSDARG00000099194 | ENSDARG00000099194 | 25 | 3521791 | 3547021 | protein\_coding |
| ENSDARG00000077089 | blm | 18 | 26444699 | 26469298 | protein\_coding |
| ENSDARG00000039616 | cenpk | 1 | 30776171 | 30783544 | protein\_coding |
| ENSDARG00000079905 | mark2a | 21 | 27167051 | 27236452 | protein\_coding |
| ENSDARG00000045853 | rad51ap1 | 4 | 5187899 | 5194773 | protein\_coding |
| ENSDARG00000042147 | rbbp5 | 22 | 732278 | 746387 | protein\_coding |
| ENSDARG00000002591 | ruvbl1 | 6 | 40835099 | 40843496 | protein\_coding |
| ENSDARG00000103907 | sgol1 | 19 | 48692230 | 48704029 | protein\_coding |
| ENSDARG00000045513 | taf3 | 4 | 25095030 | 25179260 | protein\_coding |
| ENSDARG00000058260 | zgc:174160 | 24 | 25046215 | 25088860 | protein\_coding |
